# Glymphatic system dysfunction mediates the relationship between deep medullary vein alterations and cognitive impairment in cerebral small vessel disease

**DOI:** 10.1186/s12883-025-04535-4

**Published:** 2025-12-17

**Authors:** Wenli Lu, Shengnan Zhu, Ran Chen, Li Yang, Jing Qiang, Liya Ji, Cheng Li, Dan Zhou

**Affiliations:** Department of Radiology, BenQ Medical Center, The Affiliated BenQ Hospital of Nanjing Medical University, Jianye District, No. 71 Hexi Street, Nanjing, 210019 China

**Keywords:** Cerebral small vessel disease, Deep medullary vein, Diffusion tensor imaging, Cognitive decline

## Abstract

**Background:**

This study investigates how structural changes in deep medullary vein (DMV), glymphatic system dysfunction, and cognitive decline are interconnected in cerebral small vessel disease (CSVD), with a focus on whether impaired glymphatic function acts as a mediator in this relationship.

**Methods:**

Clinical and MRI data from 93 CSVD patients were retrospectively analyzed. DMV burden was assessed using a semiquantitative scoring system (0–3 points per region), based on the visibility of DMVs in six anatomical regions on susceptibility-weighted imaging, yielding a total score ranging from 0 to 18. Glymphatic system function was evaluated using the diffusion tensor image analysis along the perivascular space (DTI-ALPS) index. Global cognitive function was assessed with the Montreal Cognitive Assessment (MoCA). Spearman correlation analysis, general linear modeling, and mediation analysis were conducted to examine the relationships among the variables.

**Results:**

DMV scores(which higher scores indicate poorer venous visibility)were significantly negatively correlated with MoCA scores (*r* = -0.48, *p*< 0.001) and with the DTI-ALPS index (*r* = -0.28, *p* < 0.001), while the DTI-ALPS index was positively correlated with MoCA scores (*r*= 0.35, *p* < 0.05). Mediation analysis indicated that the DTI-ALPS index partially mediated the effect of DMV burden on cognitive performance, accounting for 14.08% of the total effect.

**Conclusions:**

This study suggests that DMV structural abnormalities may exacerbate CSVD-related cognitive impairment by disrupting glymphatic function. DMV scoring may serve as a potential imaging biomarker, providing a foundation for early identification and intervention.

## Introduction

Cerebral small vessel disease (CSVD) refers to a group of chronic vascular pathologies affecting small arteries, venules, capillaries, and microveins within the brain. Its characteristic neuroimaging features include white matter hyperintensities (WMHs), lacunar infarcts, cerebral microbleeds, and enlarged perivascular spaces (EPVS) [1]. CSVD is one of the leading causes of vascular cognitive impairment and dementia. Its pathogenesis is complex and involves multiple pathological processes, including disruption of the blood–brain barrier, chronic hypoperfusion, neuroinflammation, and impaired clearance of metabolic waste products [2].

In recent years, the role of the venous system in CSVD has drawn increasing attention. The deep medullary vein (DMV), which serve as the primary venous drainage pathways in the white matter, radiate from the periventricular regions toward the centrum semiovale and are involved in the clearance of brain metabolic waste and interstitial fluid [3]. With advances in susceptibility-weighted imaging (SWI), the visibility of DMV has significantly improved, enabling the development of a scoring system to quantify their clarity and continuity in specific brain regions [4], This DMV scoring system has been validated as a potential imaging biomarker for white matter damage, brain atrophy, and total CSVD burden [5, 6]. Studies have shown that higher DMV scores are significantly associated with poorer global cognitive performance in CSVD patients, suggesting a potentially critical role of DMV abnormalities in the development of cognitive decline [7, 8].

On the other hand, the glymphatic system (GS), a critical pathway for clearing metabolic waste from the brain, has gained significant attention in recent years, particularly in the study of neurodegenerative diseases such as Parkinson’s disease. It facilitates convective exchange between cerebrospinal fluid (CSF) and interstitial fluid (ISF) through perivascular spaces (PVS), and its function depends on factors such as arterial pulsation, the polarized expression of aquaporin-4 (AQP4) channels, and sleep state [9]. Recently, the diffusion tensor image analysis along the perivascular space (DTI-ALPS) index has been introduced as a noninvasive MRI-based method to assess GS function. This index reflects the alignment between the orientation of PVS and white matter fiber tracts [10, 11]. Studies have shown that CSVD patients exhibit significantly lower DTI-ALPS index compared to healthy controls, and these reductions are associated with greater white matter damage, brain atrophy, and cognitive decline [12, 13]。.

Notably, there is a close anatomical relationship between the DMVs and the GS. DMV are predominantly located in the periventricular and deep white matter regions, often traversing or adjacent to the PVS—key conduits for CSF and ISF flow [14, 15]. Structural abnormalities in the DMVs may disrupt the morphology and hydrodynamics of surrounding PVS [16]. Using 7 T MRI, Zhang et al. observed that chronic venous stasis in the DMV can lead to deformation of the PVS and suppression of glymphatic clearance efficiency [17]. Animal studies have further shown that venous occlusion can result in PVS enlargement, loss of AQP4 polarity, and impaired waste clearance function [18], supporting the hypothesis that DMV abnormalities may contribute to cognitive impairment through GS dysfunction.

Although associations among DMV abnormalities, GS dysfunction, and cognitive decline have been individually reported, studies integrating these elements into a unified pathological framework remain scarce. Therefore, this study aimed to systematically evaluate the relationships among DMV burden, DTI-ALPS index, and cognitive function using multimodal MRI data, and to further explore the mediating role of the GS in the link between DMV abnormalities and cognitive impairment. This may offer a novel perspective on the mechanisms underlying cognitive decline in CSVD.

## Materials and methods

### Participants

This study enrolled 93 patients with varying levels of total CSVD burden who visited BenQ Hospital, affiliated with Nanjing Medical University, between June 2023 and December 2024. Inclusion criteria were: (1) a CSVD diagnosis based on STRIVE-2 criteria [19], with a total imaging burden score ≥ 1; (2) age ≥ 18 years; (3) right-handedness; (4) the ability to undergo MRI and complete neuropsychological assessments; and (5) provision of written informed consent. Exclusion criteria included: (1) comorbid cardioembolic stroke, intracranial large artery stenosis, or intracerebral hemorrhage; (2) non-vascular white matter lesions or cognitive impairment from non-vascular causes; (3) severe visual, auditory, or motor impairments that precluded MRI or cognitive testing; (4) diagnosed psychiatric disorders or a history of traumatic brain injury; (5) presence of brain tumors or other neurological disorders; (6) claustrophobia or other contraindications to MRI; and (7) refusal to provide informed consent.

This study was approved by the ethics committee of Affiliated BenQ Hospital of Nanjing Medical University (No. 2024-KL022) and written informed consent was obtained from all participants.

### Neuropsychological assessments

All participants completed neuropsychological assessments within one day before or after the MRI scan. Global cognitive function was evaluated using the Montreal Cognitive Assessment (MoCA) [20], which covers multiple cognitive domains including visuospatial and executive functions, naming, attention, memory, language, and orientation. The total score ranges from 0 to 30, with scores below 26 indicating cognitive impairment and scores below 18 considered indicative of dementia [21]. An additional point was added for individuals with 12 or fewer years of education [22]. The assessments were conducted in a quiet, distraction-free environment under the supervision of an experienced examiner.

### MRI protocol

All participants in this study underwent MRI scanning using a 3.0T scanner (Discovery MR750, GE Medical Systems) equipped with a 19-channel head radiofrequency coil. To minimize the influence of sleep on glymphatic function assessment, all patients were instructed to remain awake prior to the scan. The MRI protocol included the following sequences: T1-FLAIR, T2-weighted imaging (T2WI), diffusion-weighted imaging (DWI), T2-FLAIR, SWI (TR/TE = minimum/45 ms, FOV = 240 × 240 mm², slice thickness = 3 mm, flip angle = 15°, readout bandwidth = 41.67 Hz/pixel), 3D-BRAVO (TR/TE = 7.4/2.8 ms, FOV = 256 × 256 mm², matrix = 256 × 256, 190 slices, slice thickness = 1 mm, NEX = 1, flip angle = 15°, readout bandwidth = 41.67 Hz/pixel), and DTI (TR/TE = 15,033/minimum ms, matrix = 112 × 112, FOV = 224 × 224 mm², slice thickness = 2 mm, 72 slices, 64 diffusion directions, b-value = 1000 s/mm², NEX = 1). All imaging data were imported and processed using RadiAnt DICOM Viewer software.

### DTI-ALPS index

**Fig. 1:**
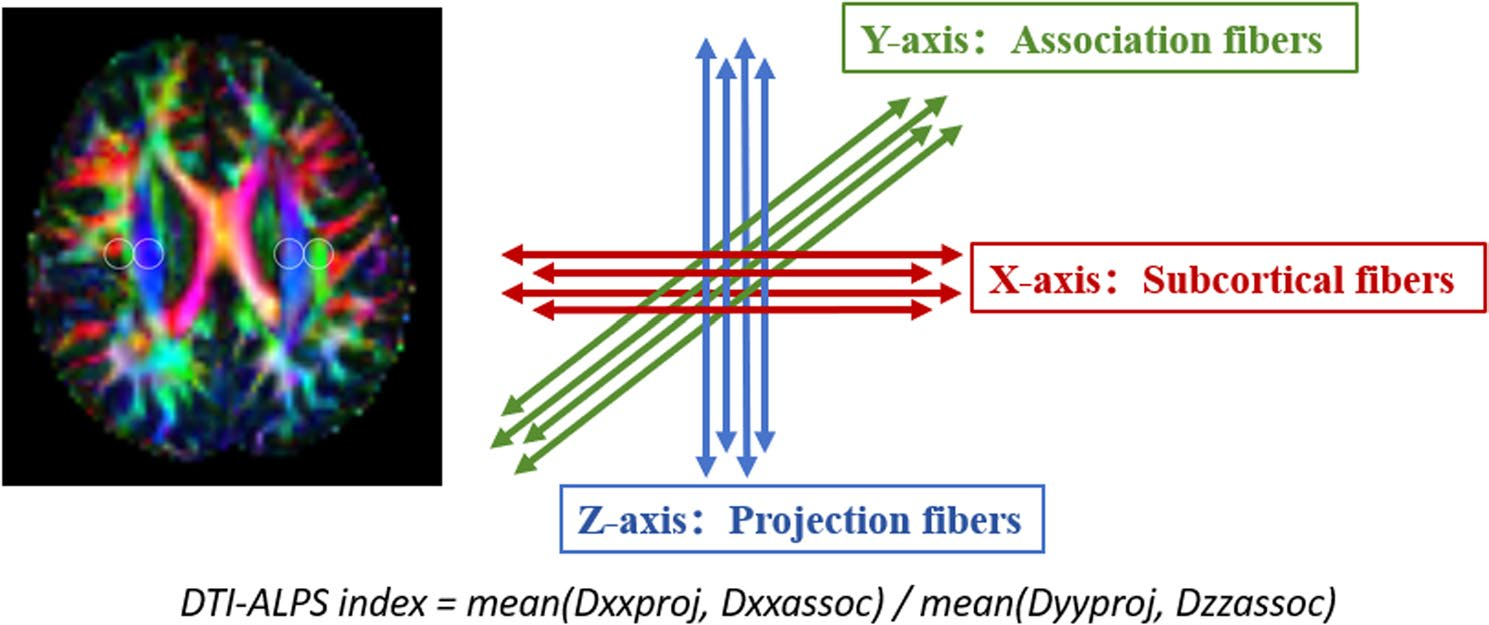
Example of regions of interest (ROIs) placement for calculating the DTI-ALPS index. On the axial fractional anisotropy color map at the level of the lateral ventricular body, the standard placement of 5-mm diameter ROIs is demonstrated within this anatomical framework, with blue areas designating projection fibers and green areas designating association fibers. ROIs, regions of interest; DTI-ALPS, diffusion tensor image analysis along the perivascular space

This study utilized diffusion tensor imaging (DTI) data and a standardized processing pipeline integrating the FMRIB Software Library (FSL) and MRtrix [11, 23] to calculate the DTI-ALPS index for assessing glymphatic system function. The workflow consisted of the following steps: (1) Brain extraction was performed on the registered b0 images to remove non-brain tissues such as the scalp and skull; (2) Affine registration and FSL’s built-in eddy current correction tools were applied to correct for head motion and eddy current-induced distortions; (3) Tensor reconstruction was conducted using FSL’s dtifit command on the preprocessed b0 images to generate fractional anisotropy (FA) and mean diffusivity (MD) maps; (4) On the color-coded FA maps at the level of the lateral ventricular body, spherical regions of interest (ROIs) with a diameter of 5 mm were manually placed in projection, association, and subcortical fiber areas in both hemispheres, and diffusion coefficients along the X-, Y-, and Z-axes were extracted from each ROI; (5) Using ImageJ, diffusion values in the X and Y directions from the projection fiber area (Dxxproj, Dyyproj) and in the X and Z directions from the association fiber area (Dxxassoc, Dzzassoc) were quantified. The standardized ALPS index was then calculated as follows: DTI-ALPS index = mean (Dyyproj, Dzzassoc)/mean (Dxxproj, Dxxassoc), with the final index taken as the average of the values from both hemispheres [24]. All spatial transformations and ROI placements were visually inspected and manually adjusted when necessary to ensure anatomical accuracy (Fig. 1).

### DVM scores

**Fig. 2:**
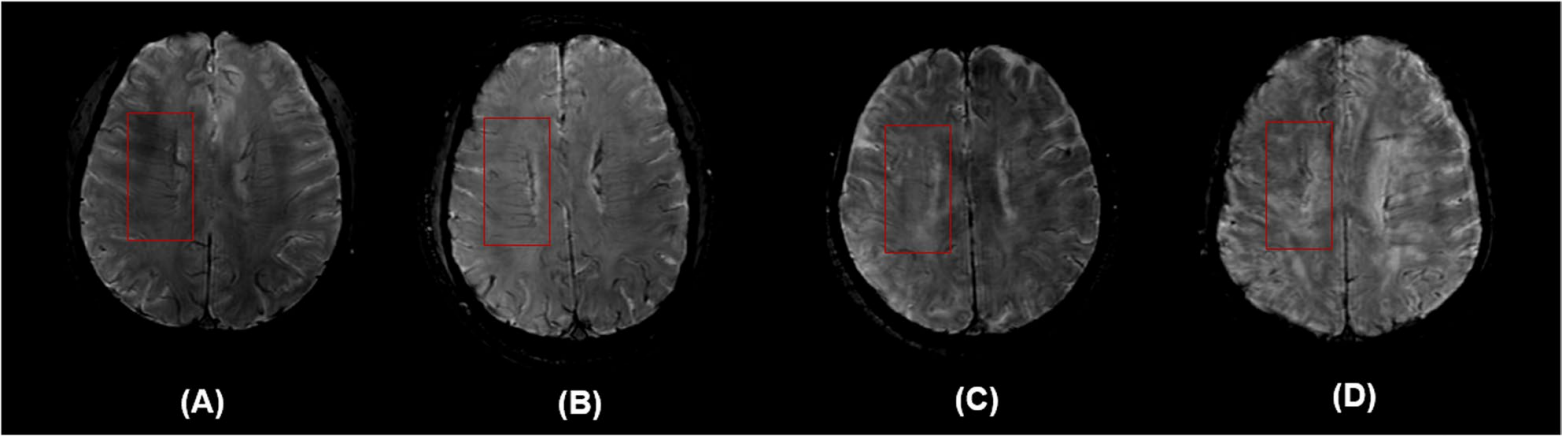
Representative images of the DMV scoring system. **A** A score of 0 indicates clearly visible and continuous DMVs; (**B**) A score of 1 indicates that at least one DMV appears discontinuous; (**C**) A score of 2 indicates faintly visible DMVs; (**D**) A score of 3 indicates that DMVs are completely invisible. DMV, deep medullary vein

To minimize observer bias, the visibility of DMVs on SWI images was independently assessed by two radiologists who were blinded to the patients’ clinical information and other imaging data. In cases of disagreement, a third experienced neuroradiologist was consulted to review the case, and a consensus was reached through discussion to determine the final DMV score. DMV scoring was performed using SWI images, selecting five periventricular slices starting at the level just above the basal ganglia. DMV scoring was based on their visibility on SWI images. According to the anatomical distribution of the DMVs, the brain was divided into six regions: the frontal, parietal, and occipital lobes of both hemispheres. Each region was scored based on the clarity and continuity of the DMV signals [5], using the following criteria: 0 points: clear and continuous DMVs; 1 point: continuous DMVs with inhomogeneous signal; 2 points: discontinuous DMVs appearing as dot-like low-signal; 3 points: DMVs not visible at all. The total DMV score was calculated by summing the scores of all six regions, ranging from 0 to 18 (Fig. 2). A higher score indicates poorer visibility of the DMVs, reflecting more severe venous alterations [6].

### Statistical analysis

Statistical analyses were performed using SPSS 26.0. continuous variables were assessed for normality distribution, with normally distributed data presented as mean ± standard deviation and non-normally distributed variables expressed as median with interquartile range (IQR). Categorical variables were reported as frequencies and percentages (%). Relationships among DMV scores, DTI-ALPS index, and MoCA scores were examined using Spearman’s correlation analysis. General linear regression modeling was conducted to identify factors influencing cognitive function in CSVD. Subsequent mediation analysis was performed using the PROCESS macro for SPSS (Version 4.1, Model 4, developed by Preacher and Hayes) [25] to examine the potential mediating role of the DTI-ALPS index between DMV scores and MoCA scores. In this model configuration, MoCA scores served as the dependent variable, DMV scores as the independent variable, and the DTI-ALPS index as the mediator. The model was adjusted for covariates including hypertension, diabetes mellitus, hyperlipidemia, and smoking history. The analysis incorporated 5,000 bootstrap resamples to estimate 95% bias-corrected confidence intervals (CIs). The indirect (mediating) effect was considered statistically significant if the 95% CI did not include zero. The effect size of the mediation was reported as the proportion of the total effect accounted for by the indirect effect.

## Results

The study cohort comprised 93 CSVD patients, including 55 males (59.1%), with a mean age of 63.29 ± 11.04 years. Comorbid conditions included hypertension in 55 cases (59.1%), diabetes mellitus in 24 cases (25.8%), and prior stroke history in 19 cases (20.4%). Imaging parameters revealed a DTI-ALPS index of 1.38 ± 0.16, DMV score median of 9 (IQR 7–12), and MoCA score median of 19 (IQR 14–26). The median CSVD total burden score was 2 (IQR 2–3). Complete clinical and imaging characteristics are presented in Table 1.

**Table 1:** General clinical, demographic, and imaging characteristics of CSVD patients

Variables	*n* = 93
Sex, male, n(%)	55 (59.1%)
Age, years	63.29 ± 11.04
Educational level, ≥ 12years, n(%)	16 (17.2%)
Hypertension, yes, n(%)	55 (59.1%)
Diabetes mellitus, yes, n(%)	24 (25.8%)
Hyperlipidemia, yes, n(%)	12 (12.9%)
History of stroke, yes, n(%)	19 (20.4%)
Smoking, yes, n(%)	39 (41.9%)
Drinking, yes, n(%)	37 (39.8%)
MoCA scores	19(14,26)
Total CSVD MR score	2 (2, 3)
DTI-ALPS index	1.38 ± 0.16
DMV scores	9 (7, 12)

Note: Values are presented as number (%) for categorical variables, mean ± SD for normally distributed continuous variables or median (interquartile range) for non-normally distributed continuous variables

*CSVD* Cerebral small vessel disease, *DMV* Deep medullary vein, *DTI-ALPS* Diffusion tensor image analysis along the perivascular space, *MoCA* Montreal cognitive assessment

### Association between DMV scores and cognitive function

**Fig. 3:**
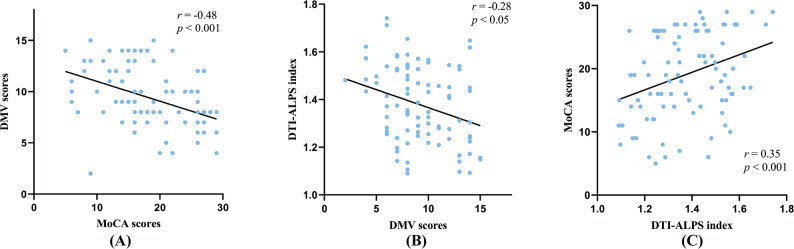
Graphs showing relationships between DMV scores, DTI-ALPS index and MoCA scores. **A** DMV scores vs. MoCA scores: Negative association (*r* = −0.48); (**B**) DMV scores vs. DTI-ALPS index: Negative association (*r* = −0.28); (**C**) DTI-ALPS index vs. MoCA scores: Positive correlation (*r* = 0.35). DMV, deep medullary vein; DTI-ALPS, diffusion tensor image analysis along the perivascular space; MoCA, montreal cognitive assessment

Spearman’s correlation analysis demonstrated a moderate negative correlation between DMV scores(which higher scores indicate poorer venous visibility) and MoCA scores (*r* = −0.48, *p* < 0.001; Fig. 3a) indicating that elevated DMV scores were significantly associated with cognitive decline. In multivariate linear regression analysis adjusted for age, vascular risk factors, and total CSVD burden score, DMV scores remained an independent predictor of MoCA scores (β = −0.259, 95% CI: −1.071 to −0.093; *p* = 0.020; Table 2).

**Table 2:** Linear regression analysis of MoCA scores in patients with CSVD

	B	β	*p* value	Β (95% CI)		VIF
				Lowest	Highest	
Age	−0.102	−0.168	0.189	−0.255	0.051	2.068
History of stroke	1.141	0.069	0.514	−2.318	4.600	1.419
Hypertension	2.011	0.148	0.133	−0.627	4.649	1.227
Diabetes mellitus	2.526	0.166	0.080	−0.305	5.358	1.120
Hyperlipidemia	−3.228	−0.162	0.105	−7.142	0.685	1.256
Smoking	1.648	0.122	0.342	−1.783	5.079	2.091
Drinking	−0.511	−0.037	0.767	−3.924	2.903	2.037
Total CSVD MR score	−1.227	−0.168	0.136	−2.848	0.394	1.591
DMV scores	−0.582	−0.259	0.020	−1.071	−0.093	1.538

*CI* Confidence interval, *CSVD* Cerebral small vessel disease, *DMV* Deep medullary vein

### Association between DMV scores and DTI-ALPS index

Subsequent analysis revealed a statistically significant negative correlation between DMV scores and the DTI-ALPS index (*r* = −0.28, *p* < 0.05; Fig. 3b) suggesting that elevated DMV scores may reflect impaired GS function.

### Association between DTI-ALPS index and cognitive function

The DTI-ALPS index demonstrated a significant positive correlation with MoCA scores (*r* = 0.35, *p* < 0.001; Fig. 3c), indicating that enhanced glymphatic system activity may be associated with better cognitive performance.

### Mediation analysis of DMV Scores, DTI-ALPS Index, and cognitive function

**Fig. 4:**
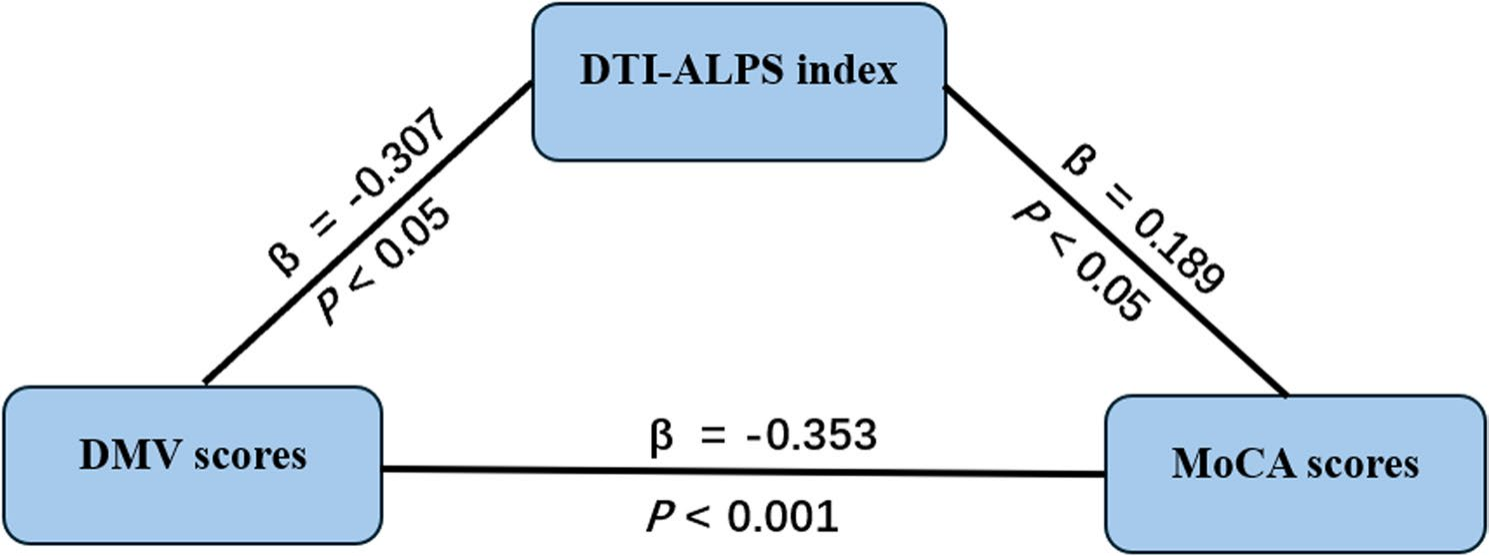
Mediation model analyses exploring relationships between DMV scores, DTI-ALPS index and MoCA scores. DMV, deep medullary vein; DTI-ALPS, diffusion tensor image analysis along the perivascular space; MoCA, montreal cognitive assessment

To investigate whether the DTI-ALPS index mediates the association between DMV scores and cognitive function, a mediation model was constructed using PROCESS macro Mode 4. The analysis demonstrated a significant indirect effect of DMV scores on MoCA scores mediated through GS function (DTI-ALPS index) after adjusting for confounders including hypertension, diabetes mellitus, hyperlipidemia, and smoking history (β = 0.189, *p* < 0.05), accounting for 14.08% of the total effect (95% CI: −0.149 to −0.001; Table 3). To comprehensively assess the directionality of the relationship, a reverse mediation model was also tested. Notably, the indirect effect of the DTI-ALPS index on MoCA scores through DMV scores was also significant and substantial, accounting for 35.28% of the total effect (Fig. 4).

**Table 3:** Total effect, direct effect and mediating effect of DMV scores on MoCA scores

	Effect size	Boot SE	Boot LLCI	Boot ULCI	Proportion of relative effect
Total effect	−0.412	0.094	−0.599	−0.224	-
Direct effect	−0.353	0.097	−0.546	−0.160	85.68%
Mediating effect	−0.058	0.038	−0.149	−0.001	14.08%

## Discussion

This multi-modal MRI study systematically investigated the relationships between DMV scores, GS function (DTI-ALPS index), and cognitive impairment in CSVD patients, while establishing the mediating role of the DTI-ALPS index in the association between DMV abnormalities and cognitive impairment. Key findings include: (1) Elevated DMV scores independently predicted worse MoCA performance after adjusting for confounders; (2) Inverse correlations between DMV scores and DTI-ALPS index; (3) GS dysfunction partially mediated DMV-cognition associations, accounting for 14.08% of total effects.

First, the moderate negative DMV scores and MoCA scores correlation (*r* = −0.48) suggests that impaired venous drainage (reflected by DMV visibility loss) may reduce metabolic waste clearance efficiency, exacerbating neural toxicity [14]. As critical white matter drainage conduits, DMV abnormalities have been linked to microstructural disintegration and atrophy [3, 6]. Our finding that DMV scores remained an independent cognitive predictor beyond traditional CSVD markers (e.g., white matter hyperintensities) highlights their unique value in risk stratification. Unlike conventional lesion-based metrics, DMV alterations may reflect early hemodynamic disturbances preceding overt tissue damage.

Second, the DMV scores and DTI-ALPS index association (*r* = −0.28) implies venous structural compromise may impair perivascular clearance. The GS relies on intact PVS and CSF dynamics [9]. Anatomically adjacent to PVS in periventricular white matter, DMV abnormalities could disrupt CSF-ISF exchange through altered venous pulsatility or PVS deformation [26]. Our findings extend prior reports [27] of DTI-ALPS index reductions in CSVD by identifying DMV alterations as a potential upstream contributor to GS.

More importantly, this study is the first to validate the mediating pathway of “DMV structural abnormalities - GS dysfunction - cognitive impairment” pathway in CSVD, supporting the role of impaired clearance mechanisms in CSVD-related cognitive impairment. Mediation analysis showed that the DTI-ALPS index partially mediated the relationship between DMV scores and MoCA scores. This finding suggests that DMV abnormalities may contribute to cognitive decline by weakening the brain’s clearance system, thereby promoting the accumulation of neurotoxic metabolites such as amyloid-β and phosphorylated tau, and accelerating neurodegenerative processes [28, 29]. However, the mediation effect accounted for only 14.08% of the total effect, indicating that, beyond the glymphatic system, DMV-related cognitive impairment may involve other mechanisms, such as white matter structural damage [30], neurotoxic responses due to hypoperfusion [31], or disrupted brain network connectivity [32]. These factors likely interact to form a multifactorial network underlying cognitive dysfunction in CSVD.

The clinical significance of this study lies in the use of non-invasive MRI-derived markers with potential value for early prediction and subtyping of cognitive impairment in CSVD patients. These markers may aid in the early identification of individuals at higher risk for cognitive decline. Moreover, the findings provide theoretical support for future therapeutic strategies targeting the brain’s clearance system, such as restoration of AQP4 function and enhancement of cerebrospinal fluid dynamics [33]. An important finding was the significant mediation effect also observed in the reverse model, with glymphatic function as the independent variable and DMV score as the mediator. This suggests a close, potentially bidirectional interaction between the venous and glymphatic systems in CSVD. Although both directional relationships are statistically supported, we propose that the sequence “structural venous abnormality → glymphatic dysfunction” represents the more dominant initiating pathological pathway. This view is consistent with the anatomical basis by which venous structural impairment can mechanically disrupt PVS hydrodynamics, as well as experimental evidence showing that venous occlusion induces glymphatic impairment. Nonetheless, the significant reverse pathway suggests the potential existence of a secondary, reinforcing vicious cycle, wherein impaired clearance and neuroinflammation may in turn exacerbate venous injury.

However, several limitations should be noted. First, the study employed a single-center, retrospective, cross-sectional design, which limits the ability to establish causal relationships. The finding of significant indirect effects in both tested mediation models underscores the complex and likely bidirectional relationship between DMV integrity and glymphatic function. While we posit, based on current theoretical and experimental literature, that the DMV → DTI-ALPS pathway is more plausible, this specific causal sequence requires further validation through longitudinal studies or animal models capable of independently manipulating one of these systems. Second, the sample size was relatively small, and some potential confounding factors—such as sleep quality, time of day for MRI acquisition, and AQP4 genotype—were not controlled. Third, the DTI-ALPS index, although useful, is an indirect marker and may be influenced by imaging resolution and ROI placement variability. Furthermore, the DMV scoring system, based on SWI, may be subject to observer variability despite efforts to minimize bias. Future studies employing higher-resolution imaging (e.g., 7 T MRI) and multimodal assessments (e.g., DCE-MRI, ASL) are warranted.

## Conclusions

This study elucidates a novel “DMV structural abnormalities - GS dysfunction - cognitive impairment” pathway in CSVD pathophysiology. DMV scores and DTI-ALPS index emerge as quantifiable imaging biomarkers for early cognitive risk identification and therapeutic targeting. Our findings provide preliminary insights into possible mechanisms underlying CSVD-related neurodegeneration while providing a framework for developing glymphatic-focused interventions.

## Data Availability

The datasets generated during this study are available from the corresponding author on reasonable request. Public sharing is restricted to protect participant confidentiality in accordance with ethical guidelines.

## Abbreviations

AQP4: Aquaporin-4
BG-EPVS: Enlarged perivascular spaces in the basal ganglia
CIs: Confidence intervals
CSF: Cerebrospinal fluid
CSVD: Cerebral small vessel disease
DWI: Diffusion-weighted imaging
DMV: Deep medullary vein
DTI-ALPS: Diffusion tensor image analysis along the perivascular space
EPVS: Enlarged perivascular spaces
FA: Fractional anisotropy
GS: Glymphatic system
IQR: Interquartile range
ISF: Interstitial fluid
MD: Mean diffusivity
MoCA: Montreal cognitive assessment
PVS: Perivascular spaces
ROIs: Regions of interest
SWI: Susceptibility-weighted imaging
T2WI: T2-weighted imaging
WMHs: White matter hyperintensities
